# A putative multi-sensor hybrid histidine kinase, BarA*_Ac_*, inhibits the expression of the type III secretion system regulator HrpG in *Acidovorax citrulli*

**DOI:** 10.3389/fmicb.2022.1064577

**Published:** 2022-11-30

**Authors:** Pei Qiao, Mei Zhao, Wei Guan, Ron Walcott, Yunfeng Ye, Yuwen Yang, Tingchang Zhao

**Affiliations:** ^1^State Key Laboratory for Biology of Plant Diseases and Insect Pests, Institute of Plant Protection, Chinese Academy of Agricultural Sciences, Beijing, China; ^2^Department of Plant Pathology, College of Plant Protection, China Agricultural University, Beijing, China; ^3^Department of Plant Pathology, University of Georgia, Athens, GA, United States; ^4^Horticultural Research Institute, Guangxi Academy of Agricultural Sciences, Nanning, China

**Keywords:** *barA_Ac_*, virulence, HrpG, regulation, T3SS, expression

## Abstract

Bacterial fruit blotch (BFB), caused by *Acidovorax citrulli*, severely damages watermelon, melon, and other cucurbit crops worldwide. Although many virulence determinants have been identified in *A. citrulli*, including swimming motility, twitching motility, biofilm formation, and the type III secretion system (T3SS), research on their regulation is lacking. To study virulence regulation mechanisms, we found a putative histidine kinase BarA*_Ac_* that may be related to the T3SS regulator HrpG in *A. citrulli*. We deleted and characterized *barA_Ac_* (*Aave_2063*) in *A. citrulli* Aac5 strain. Compared to the wild-type Aac5, virulence and early proliferation of *barA_Ac_* mutant in host watermelon cotyledons were significantly increased, and induction of hypersensitive response in non-host tobacco was accelerated, while biofilm formation and swimming motility were significantly reduced. In addition, the transcriptomic analysis revealed that the expression of many T3SS-related genes was upregulated in the Δ*barA_Ac_* deletion mutant when cultured in KB medium. Meanwhile, the Δ*barA_Ac_* deletion mutant showed increased accumulation of the T3SS regulator HrpG in KB medium, which may account for the increased deployment of T3SS. This suggests that the putative histidine kinase BarA*_Ac_* is able to repress the T3SS expression by inhibiting HrpG in the KB medium, which appears to be important for rational energy allocation. In summary, our research provides further understanding of the regulatory network of *A. citrulli* virulence.

## Introduction

Bacterial fruit blotch (BFB) is an economically important disease of watermelon, melon, and other cucurbit species. Since BFB was first reported in 1965, it has occurred in watermelon and melon producing areas all over the world (Webb and Goth, 1965; Latin, 1990; Ren et al., 2006; Song et al., 2015), and has been increasing each year in China and sporadically in other countries (Zhao et al., 2001; Zhao and Walcott, 2018; Fei et al., 2022). The causal agent of BFB is the gram-negative bacterium *Acidovorax citrulli* (Schaad et al., 2008) and strains of the pathogen can be divided into two major groups (I and II; Walcott et al., 2004). Field experiments conducted under natural conditions strongly reveal that *A*. *citrulli* strains manifest host preferential association between the two groups of strains (Zhao et al., 2020). *Acidovorax citrulli* can be disseminated through the international trade and movement of cucurbit seeds, and all developmental stages of the host plant can be infected by the bacterium (Walcott et al., 2003). Great efforts have been expended to mitigate the impact of BFB, but unfortunately, the efficacy of the current management strategies has been limited and BFB still poses a serious threat to commercial cucurbit production worldwide (Burdman and Walcott, 2012).

At present, researchers have identified several virulence factors of *A. citrulli*, including swimming motility (Bahar et al., 2011; Zhang et al., 2017; Guan et al., 2020), twitching motility (Bahar et al., 2009; Rosenberg et al., 2018), biofilm formation (Bahar et al., 2010; Wang et al., 2016), the type III secretion system (T3SS; Ren et al., 2009; Wang et al., 2011; Yan et al., 2015; Zhang et al., 2018, 2020a,b; Yu et al., 2019), etc. Among them, T3SS plays an important role in bacterial virulence (Johnson et al., 2011). Many plant pathogenic bacteria can inject effectors directly into host plant cells *via* the T3SS (Lewis et al., 2013; Dortet et al., 2018). Without a T3SS, many plant pathogenic bacteria cannot successfully colonize the host due to their inability to overcome basic plant host resistance (Alfano and Collmer, 1997). The genes encoding T3SS components are called hypersensitive response (HR) and pathogenicity (*hrp*) genes (Kim et al., 2003; Tampakaki et al., 2010). At present, according to gene organization, sequence, and regulation, *hrp* clusters can be divided into two classes: class I contains clusters of *Pseudomonas syringae* and *Erwinia amylovora*, and class II contains *hrp* genes of *Xanthomonas campestris* and *Ralstonia solanacearum* (Bogdanove et al., 1996; Büttner and Bonas, 2002). The *A. citrulli hrp* cluster belongs to class II (Burdman and Walcott, 2012). A recent study (Zhang et al., 2018) suggested that *hrpG* was the key regulatory gene of T3SS in *A. citrulli*. HrpG is an OmpR-type regulator capable of activating the transcription of AraC-type activator *hrpX* (Wengelnik et al., 1996; Wengelnik and Bonas, 1996; Wengelnik et al., 1999). The deletion of *hrpG* affected the expression of downstream T3SS-related genes including *hrpX*, resulting in loss of pathogenicity of *A. citrulli* (Zhang et al., 2018). Notably, induction of the *hrpG*/*hrpX* regulon is mediated by sensing the plant environment (Teper et al., 2021). Similar to several other phytopathogens using *hrp*-T3SS, the *hrp* genes of *A. citrulli* were suppressed in rich media such as King’s B broth (KB), but induced in plant environment or medium that simulates plant environment such as XVM2 medium (Zhang et al., 2018).

Histidine kinases belong to a component of a two-component system (TCS). It can help microorganisms quickly capture and sense external chemical or physical signals, and adapt to the unpredictable external environment by phosphorylating response regulators in the TCS to respond accordingly. Many histidine kinases and their response regulators are involved in the growth and virulence of bacteria, and some of them affect virulence mainly through regulation (Osteras et al., 1995; Heeb and Haas, 2001; Navarre et al., 2005; Pratt et al., 2010; Zhao et al., 2012). Willis et al. (1990) identified for the first time the role of the histidine kinase GacS (or named BarA) in regulating virulence of *Pseudomonas syringae*, and GacS homologs have since been shown to modulate the virulence of many pathogens, including *Vibrio cholerae*, *Salmonella typhimurium*, *Pseudomonas aeruginosa*, and other gram-negative bacteria (Johnston et al., 1996; Wong et al., 1998; Heeb and Haas, 2001; Valentini et al., 2017). GacS and its homologs can phosphorylate the response regulator GacA to negatively regulate the core regulatory genes of T3SS and affect the deployment of the entire T3SS in *P. aeruginosa* and *P. syringae* (Valentini et al., 2017; O'Malley et al., 2020). However, there are few reports about the involvement of GacS/GacA in the regulation of class II-Hrp T3SS. GacA in *Xanthomonas oryzae* pv. *oryzae* was reported to regulate swimming motility but not T3SS (Yang, 2006; Xu, 2007).

Currently, there are no reports on the regulation of T3SS regulators in *A. citrulli*. So, is there regulator in *A*. *citrulli* similar to that found in *P. syringae* in class I-Hrp that participates in the regulation of T3SS? We found the gene (*Aave_2063*; hereafter named *barA_Ac_*) encoding a multi-sensor hybrid histidine kinase in *A. citrulli* Aac5 strain is a homolog of histidine kinase BarA. STRING (a website about functional protein association networks) analysis shows HrpG may interact with the protein encoded by *Aave_2063*. Characterizing *barA_Ac_* may broaden our understanding of T3SS regulatory pathways in *A. citrulli*. The objectives of this study were to characterize the role of *barA_Ac_* in *A. citrulli* Aac5 in virulence associated phenotypes, and its role in virulence regulation.

## Materials and methods

### Sequence and phylogenetic analysis of BarA*_Ac_*

The conserved domains of BarA*_Ac_* in *A*. *citrulli* AAC00-1 and GacS in *P*. *syringae* pv. *tomato* DC3000 were analyzed using NCBI CD-search,^1^ and the STRING database.^2^ The amino acid sequences of BarA*_Ac_* of *A. citrulli* AAC00-1 and GacS of *P. syringae* pv. *tomato* DC3000 were aligned using CLUSTALW.^3^ In order to analyze the evolutionary relationship between the multi-sensor hybrid histidine kinase BarA*_Ac_* encoded by *Aave_2063* in *A*. *citrulli* AAC00-1 and the GacS (or named BarA) in several other species, their amino acid sequences were obtained from KEGG^4^ (Supplementary Table 1). Amino acid sequences were aligned using ClustalW in MEGA 7 software (Kumar et al., 2016). The phylogenetic tree was generated using the neighbor-joining method and the evolutionary distances were computed using the JTT matrix-based model (Jones et al., 1992) with the MEGA 7 software. The bootstrap values were calculated with 1,000 replicates.

### Bacterial strains, plasmids, growth conditions, and primer design

The bacterial strains and plasmids used in this study are listed in Table 1. *Acidovorax citrulli* group II strain, Aac5, was grown in KB (King et al., 1954) or agar (KBA; KB containing 15 g/L agar) at 28°C. *Escherichia coli* strains were grown in Luria Bertani medium (10 g tryptone, 5 g yeast extract, and 10 g NaCl, 1,000 ml deionized water) at 37°C. When required, media were supplemented with ampicillin (Ap) at 100 μg/ml and kanamycin (Km) at 50 μg/ml. The primer pair, 2063-S/A, was designed based on the *Aave_2063* gene in the AAC00-1 genome (GenBank accession number CP000512.1), and the primer pairs, 2063 l-S/A and 2063R-S/A, were designed based on the upstream and downstream flanking sequences of the *Aave_2063* gene (Table 2). All primers used in this study were designed using Primer 3.0.^5^

**Table 1:** Strains and plasmids used in this study.

Strain or plasmid	Description	References or sources
**Strains**		
*Escherichia coli*		
DH5α	*supE44* Δ*lacU169*(Φ80*lacZ* Δ*M15*) *hsdR17 recA1 endA1 gyrA96 thi-1 relA1*	Hanahan (1983)
*Acidovorax citrulli*		
Aac5	Wild-type watermelon strain; Ap^r^	Yan et al. (2013)
Δ*barA_Ac_*	*barA_Ac_* mutant, Ap^r^	This study
Δ*barA_Ac_*comp	Δ*barA_Ac_* complementation strain, Δ*barA* containing pBBR-*Aave_2063*; Ap^r^, Km^r^	This study
WT-pB	Aac5 containing pBBR1MCS-2; Ap^r^, Km^r^	This study
Δ*barA_Ac_*-pB	Δ*barA_Ac_* containing pBBR1MCS-2; Ap^r^, Km^r^	This study
WT-EV	Aac5 containing pBBRNolac-4FLAG; Ap^r^, Km^r^	This study
WT-*hrpG*	Aac5 containing pBBR-4FLAG-*hrpG*; Ap^r^, Km^r^	This study
Δ*barA_Ac_*-*hrpG*	Δ*barA_Ac_* containing pBBR-4FLAG-*hrpG*; Ap^r^, Km^r^	This study
WT-*hrpX*	Aac5 containing pBBR-4FLAG-*hrpX*; Ap^r^, Km^r^	This study
Δ*barA_Ac_*-*hrpX*	Δ*barA_Ac_* containing pBBR-4FLAG-*hrpX*; Ap^r^, Km^r^	This study
WT-GUS	Aac5 containing pBBRNolacGUS; Ap^r^, Km^r^	This study
WT-*hrpG*p-GUS	Aac5 containing pBBR-GUS-*hrpG*p; Ap^r^, Km^r^	This study
Δ*barA_Ac_*-*hrpG*p-GUS	Δ*barA_Ac_* containing pBBR-GUS-*hrpG*p; Ap^r^, Km^r^	This study
WT-*hrpX*p-GUS	Aac5 containing pBBR-GUS-*hrpX*p; Ap^r^, Km^r^	This study
Δ*barA_Ac_*-*hrpX*p-GUS	Δ*barA_Ac_* containing pBBR-GUS-*hrpX*p; Ap^r^, Km^r^	This study
**Plasmids**		
pK18*mobsacB*	Cloning and suicide vector with *sacB* for mutagenesis; Km^r^	Schäfer et al. (1994)
pK18-*Aave_2063*-Up&Down	pK18*mobsacB* carrying 563-bp upstream and 550-bp downstream sequences of *Aave_2063*; Km^r^	This study
pBBR1MCS-2	Broad-host range expression vector; Km^r^	Kovach et al. (1994)
pBBR-*Aave_2063*	pBBR1MCS-2 carrying 5,534-bp coding region of *Aave_2063*; Km^r^	This study
pBBRNolac-4FLAG	*lac* promoter was deleted from pBBR1MCS-2 and C-terminal 4 × FLAG tag was inserted; need the native promoter to drive expression; Km^r^	Zhang et al. (2018)
pBBR-4FLAG-*hrpG*	pBBRNolac-4FLAG carrying 1,336-bp *hrpG* sequence *of A. citrulli*; Km^r^	This study
pBBR-4FLAG-*hrpX*	pBBRNolac-4FLAG carrying 1,991-bp *hrpX* sequence *of A. citrulli*; Km^r^	This study
pBBRNolacGUS	*lac* promoter was deleted from pBBR1MCS-2 and GUS reporter gene was inserted; Km^r^	Zhang et al. (2018)
pBBR-GUS-*hrpG*p	pBBRNolacGUS carrying 468-bp promoter sequence of *hrpG*; Km^r^	This study
pBBR-GUS-*hrpX*p	pBBRNolacGUS carrying 486-bp promoter sequence of *hrpX*; Km^r^	This study
pRK600	Helper strain used in tri-parental mating; Cm^r^	Lab collection

Km^r^, Ap^r^, and Cm^r^ indicate resistant to kanamycin, ampicillin, and chloramphenicol, respectively.

**Table 2:** Primers used in this study.

Primers	Sequence (5′ → 3′)	PCR amplicon (bp)	Description
2063 l-S	CTATGACATGATTACGAATTCCATCACCACCCCCGTCAT	563	Amplification of *barA_Ac_* upstream fragment
2063 l-A	TCGAAATCGAAGTCGCGCCAAGGCGGCTACAGGAACA		
2063R-S	TGTTCCTGTAGCCGCCTTGGCGCGACTTCGATTTCGA	550	Amplification of *barA_Ac_* downstream fragment
2063R-A	ACGACGGCCAGTGCCAAGCTTCCTTCAGCGTCAGGTGGG		
2063-S	GACTACGTCGCCAAGATC	1,106	Verification of tested strains
2063-A	TGGTTCAGTTCGTTGTCC		
*Aave_2063*c-S	GGCGGCCGCTCTAGAACTAGTGCCGTGAGGTTGTCTTCCA	5,534	Amplification of *barA_Ac_* complementing sequence
*Aave_2063*c-A	GGTACCGGGCCCCCCCTCGAGATCGGAGAGCAGCGAAGTG		
F-*hrpG*-S	CGCTCTAGAACTAGTGGATCCCGATGCGTTGGGAGGAGA	1,336	Amplification of *hrpG*
F-*hrpG*-A	GGTAAGCTTGATATCGAATTCACTGCCCAGGGGCGGCAT		
F-*hrpX*-S	CGCTCTAGAACTAGTGGATCCGACTCGCATGATTTCCCCA	1,991	Amplification of *hrpX*
F-*hrpX*-A	GGTAAGCTTGATATCGAATTCGTGCCGCATCGACGACAG		
G-*hrpG*p-S	CGCGGTGGCGGCCGTGTGGTTTGGATGACGACC	468	Amplification of *hrpG* promoter
G-*hrpG*p-A	CATAAGCTTGATATCGATTTCCCCATACGCAAAC		
G-*hrpX*p-S	CGCGGTGGCGGCCGCGCAAACGTTTTCGTTGA	486	Amplification of *hrpX* promoter
G-*hrpX*p-A	CATAAGCTTGATATCTTCGATCGTCGCGGCCCC		

### Molecular manipulations

The *barA_Ac_* gene was deleted using the homologous double recombination approach, as previously described (Zhang et al., 2018). In short, the wild-type Aac5 strain was cultured in KB medium at 220 revolutions per minute (RPM) and at 28°C for 12 to 14 h. Genomic DNA was isolated from the bacterial suspension of Aac5 strain using AxyPrep™ Multisource Genomic DNA Miniprep Kit (Axygen, United States), and quantified by NanoVue Plus (GE Healthcare, United States). The 563 bp upstream and 550 bp downstream flanking sequences of the *barA_Ac_* gene were amplified from wild-type Aac5 genomic DNA using KOD-Plus-Neo (TOYOBO, Japan), the 2063-S/2063-A and 2063R-S/2063R-A primers (Table 2). The PCR fragments were fused by overlapping PCR and ligated into pK18*mobsacB* using ClonExpress II One Step Cloning Kit (Vazyme, China) to create the plasmid pK18-*Aave_2063*-Up&Down (Table 1). Then, using pRK600 as a helper plasmid, the pK18-*Aave_2063*-Up&Down was introduced from *E. coli* DH5α (TianGen, China) into *A. citrulli* Aac5 strain through tri-parental mating to create the *Aave_2063* mutant strain Δ*barA_Ac_* (Table 1). Transconjugants grown on KBA supplemented with 10% sucrose and Ap antibiotics were screened. The primers, 2063-S and 2063-A, were used to confirm the deletion of the *barA_Ac_* gene (Table 2). In order to generate a complementary strain of Δ*barA_Ac_*, primers *Aave_2063*c-S and *Aave_2063*c-A were used to amplify the *barA_Ac_* gene and the upstream 585 bp sequence in Aac5 (Table 2). The PCR product was cloned into pBBR1MCS-2 to generate pBBR-*Aave_2063*, which was transferred to the mutant strain Δ*barA_Ac_* through tri-parental mating (Table 1). By screening colonies grown on KBA (amended with Ap and Km; Table 1), a transconjugant named ∆*barA_Ac_*comp was identified. Additionally, in order to eliminate the influence of the plasmid on the bacterial host cells, pBBR1MCS-2 was transferred to wild-type Aac5 strain and the Δ*barA_Ac_* strain by tri-parental mating (Table 1). Through the screening of colonies grown on KBA (amended with Ap and Km; Table 1), successful transconjugants were identified, and named WT-pB and Δ*barA_Ac_*-pB. All plasmids and *A. citrulli* strains were confirmed by PCR and DNA sequencing (Beijing Liuhe BGI Co., Ltd., China).

### Assays for virulence, proliferation ability in host, and HR in tobacco

#### Watermelon seed transmission assay

The seed to seedling transmission of the tested *A. citrulli* strains was determined by inoculating and germinating watermelon seeds (*Citrullus lanatus* cv. “Jingxin#6,” provided by the Beijing Academy of Agriculture and Forestry Sciences, Beijing, China) following the protocol described by Zhao and Walcott (2020) with few modifications. The BFB disease index was determined as previously described (Song et al., 2020). Briefly, tested strains were cultivated in KB, resuspended in sterilized distilled water, and their absorbances were adjusted to OD_600_ = 0.3 (3 × 10^8^ CFU/ml) by spectrophotometry, and then the concentrations of the bacterial suspensions were adjusted to 3 × 10^5^ CFU/ml with sterilized distilled water. Watermelon seeds were soaked in bacterial cell suspensions at room temperature in a rotary shaker (60 rpm) for 2 h. After that, the inoculated seeds were air-dried for 24 h, and sown in plastic pots filled with potting mix (vermiculite: nutrient soil = 1:3), with 4 seeds per pot. The seedlings were cultivated for 18 days with a 16 h light/8 h dark cycle and average relative humidity of 65%. The BFB severity was evaluated by measuring the disease index 18 days after sowing (Song et al., 2020). This experiment was conducted three times.

#### Watermelon cotyledon proliferation assay

The ability of the tested strains to colonize plant host tissues was determined by quantifying the bacterial population in watermelon cotyledons as previously described (Johnson et al., 2011). Briefly, tested strains were cultivated in KB to the log phase, then resuspended in sterile water, their absorbance was adjusted to OD_600_ = 0.3 (3 × 10^8^ CFU/ml) by spectrophotometry, and diluted to 3 × 10^4^ CFU/ml with sterile water. Bacterial suspensions were injected into 2-week-old watermelon cotyledons using disposable needleless syringes. At 1, 24, 48, 72, and 96 h post inoculation (hpi), three cotyledons inoculated with the bacterial suspension were taken, and two leaf discs (1 cm diameter) were taken from each cotyledon. Afterward, the leaf discs were disinfected with 75% alcohol, washed with sterile water, ground, diluted, and spotted on KB agar. The plates were incubated at 24°C for 48 h, and the populations of the tested strains were counted. The experiment was conducted three times.

#### Tobacco HR assay

The tested strains were cultured in KB, resuspended in sterilized water, and the OD_600_ was adjusted to 0.3 using a spectrophotometer. A needleless syringe was used to infiltrate bacterial cell suspensions of different strains into tobacco leaves. At 0.5, 3, 6 and 9 hpi, a leaf disc (0.7 cm diameter) was taken from the infiltrated leaves. The discs were stirred continuously for 30 min in a beaker with 10 ml deionized H_2_O, and a DDSJ-318 conductivity meter (INESA, China) was used to measure the conductivity. The infiltrated leaves were photographed at 24 hpi. The above experiments were conducted three times.

### Assay for biofilm formation and swimming motility

The effect of *barA_Ac_* on biofilm was qualitatively and quantitatively determined as previously described (Bahar et al., 2009). In short, the overnight cultures grown in KB and XVM2 media were adjusted to OD_600_ = 0.3. One milliliter of each bacterial solution was added into a 24-well polystyrene plate, and incubated in the dark at 28°C for 48 h. Then, the bacterial solution was discarded and the plates were fixed at 80°C for 30 min, and washed with sterilized water three times. One milliliter of 0.1% crystal violet was added to the plates. Then, the plates were incubated at room temperature for 45 min, and then rinsed with sterilized water. Pictures were taken after drying the plates at 37°C. Then 1 ml of 95% ethanol was added to elute the stained biofilm, and the OD_575_ value of the eluate was measured using a spectrophotometer to analyze the biofilm-forming ability of each strain. Each treatment has six technical repetitions, and the experiment has three biological repetitions.

The swimming motility of *A. citrulli* strains was measured according to Wang et al. (2016). The test strains were cultured in KB broth and their OD_600_ values were adjusted to 0.3. Three microliters of each cell suspension were placed at the center of a 0.3% water agar medium plate (0.03% tryptone, 0.03% yeast extract, and 0.3% agar). Plates were cultured at 28°C for 48 h and then the diameter of each colony was measured. The experiment was conducted three times.

### RNA-seq library construction, sequencing, and data analysis

*Acidovorax citrulli* Aac5 and Δ*barA_Ac_* were grown in KB broth at 28°C overnight to an OD_600_ of 0.6. RNA extraction, RNA sequencing library construction and sequencing were performed by Novogene Co., Ltd. (Beijing, China). The libraries were prepared using the NEBNext Ultra RNA Library Prep Kit (NEB, United States) for Illumina, and index codes were added to attribute sequences to each sample. The clustering of the index-coded samples was performed on a cBot Cluster Generation System using a TruSeq PE Cluster Kit v. 3-cBot-HS (Illumina). After cluster generation, the libraries were sequenced on an Illumina HiSeq 2500 platform, and 100-bp paired-end reads were generated.

The sequencing data were analyzed commercially by Novogene Co., Ltd. Briefly, differential gene expression analysis between the wild-type strain Aac5 and Δ*barA_Ac_* (three biological replicates per strain) was performed using the R package DESeq (v. 1.10.1), which uses a model based on the negative binomial distribution (Anders and Huber, 2012). The resulting *p-*values were adjusted using the Benjamini and Hochberg’s approach for controlling the false discovery rate. Genes with an adjusted *p*-value < 0.05 found by DESeq were assigned as differentially expressed (Pan et al., 2018). Gene Ontology (GO) analysis of differentially expressed genes was implemented by the R package clusterProfiler (v. 3.8.1), in which gene length bias was corrected (Yu et al., 2012). GO terms with corrected *p-*value < 0.05 were considered significantly enriched. Kyoto Encyclopedia of Genes and Genomes (KEGG) is a database resource for understanding high-level functions and utilities of the biological system. R package clusterProfiler was used to test the statistical enrichment of differentially expressed genes in KEGG pathways (Yu et al., 2012).

### Gene expression analysis by quantitative reverse transcription PCR

RNA was isolated from the *A. citrulli* Aac5 and Δ*barA_Ac_* strains using the method described in Wang et al. (2016). Briefly, the tested strains were grown in KB broth at 28°C overnight to an OD_600_ of 0.6. Total *A. citrulli* RNA was extracted using the bacterial RNA kit (TransGen, China), and the cDNA was synthesized using a FastQuant RT kit (TianGen, China). cDNA was used as a template and SYBR Green (TianGen, China) was used in the PCR. Quantitative reverse transcription (RT-qPCR) analysis was carried out in a real-time PCR machine (M × 3000P, Agilent) using the following program: 95°C for 15 min (1 cycle); 95°C for 10 s, 55°C for 20 s, 72°C for 32 s (40 cycles); melting curve profiled from 55°C to 95°C to check the specificity of the reaction. The primers used in the assay are listed in Supplementary Table 2. Relative gene expression levels were determined as previously described (Wang et al., 2016). Each sample was tested three times per experiment, and the experiment was conducted three times.

### HrpG and HrpX expression assay

In order to determine the expression levels of HrpG and HrpX in the tested strains by Western blot (Towbin et al., 1979; Burnette, 1981), we used primer pairs F-*hrpG*-S/F-*hrpG*-A, F-*hrpX*-S/F-*hrpX*-A to amplify the *hrpG* and *hrpX* genes in *A. citrulli*, and ligate them to pBBRNolac-4FLAG to construct pBBR-4FLAG-*hrpG* and pBBR-4FLAG-*hrpX* (Tables 1, 2). These plasmids were transferred to Aac5 and mutant strain Δ*barA_Ac_* through tri-parental mating (Table 1). By screening colonies grown on KBA (amended with Ap and Km; Table 1), transconjugants were identified and named WT-*hrpG*, Δ*barA_Ac_*-*hrpG*, WT-*hrpX*, and Δ*barA_Ac_*-*hrpX*. pBBRNolac-4FLAG was transferred to *A. citrulli* Aac5 strain to serve as a negative control and named WT-EV. All obtained plasmids and *A. citrulli* strains were confirmed by PCR and DNA sequencing. The test strains were cultured in KB broth to OD_600_ = 0.6, and the total protein was extracted by centrifugation. At the same time, bacterial suspension (OD_600_ = 0.6, 500 μl) was centrifuged at 10,500 rpm for 1 min. Then, the supernatant was discarded, and the bacteria was resuspended with 5 ml XVM2 and cultured at 28°C with shaking for 24 h. After adjusting OD_600_ = 0.6, the total protein was extracted by centrifugation (Zhang et al., 2018). The total protein of the tested strain was separated by 10% SDS PAGE and transferred to a nylon membrane for Western blot analysis using the DDDDK antibody (MBL Beijing Biotech Co., LTD, China). RNA polymerase β was used as a reference protein and the antibody was from Biolegend (United States). The experiment was conducted three times.

### Assay for *hrpG* and *hrpX* promoter activity

We used primer pairs G-*hrpG*p-S/G-*hrpG*p-A and G-*hrpX*p-S/G-*hrpX*p-A to amplify the *hrpG* and *hrpX* promoter regions in *A. citrulli* Aac5 strain, and ligate them to pBBRNolacGUS to construct pBBR-GUS-*hrpG*p and pBBR-GUS-*hrpX*p (Tables 1, 2). Plasmids were transferred to Aac5 and Δ*barA_Ac_* through tri-parental mating (Table 1). Through screening colonies grown on KBA (amended with Ap and Km; Table 1), transconjugants were identified and named WT-*hrpG*p-GUS, Δ*barA_Ac_*-*hrpG*p-GUS, WT-*hrpX*p-GUS, and Δ*barA_Ac_*-*hrpX*p-GUS. The pBBRNolacGUS was transferred to the Aac5 strain as a negative control and named WT-GUS. All plasmids and *A. citrulli* strains were confirmed by PCR and DNA sequencing. The promoter activity was tested as previously described (Zhang et al., 2013), and this experiment was conducted three times.

### Statistical analysis

Quantitative data were analyzed using the commercial SPSS 20.0 (IBM, United States) software package. Analysis of variance (ANOVA; Duncan’s multiple range test, *p* < 0.05) was used to test the significance of multiple comparisons of different treatments, and the bar chars were made using GraphPadPrism7 (GraphPad Software, United States).

## Results

### Bioinformatic information, deletion, and complementation of *barA_Ac_*

**Figure 1:**
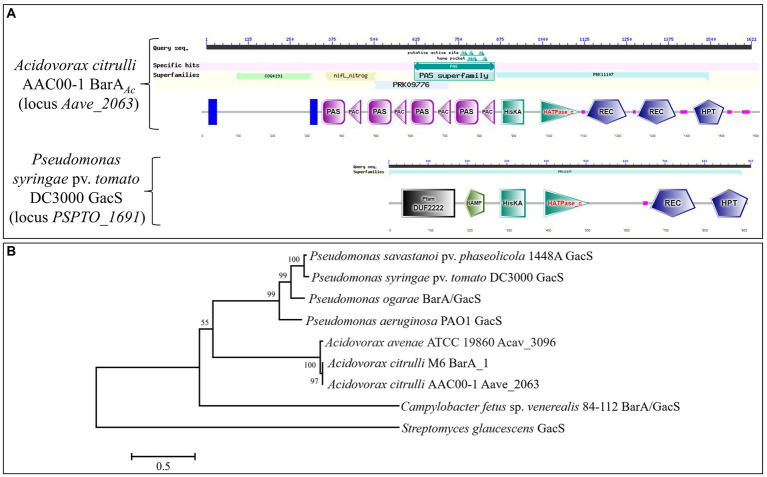
Domains of BarA and the phylogenetic tree of BarA proteins. **(A)** The domain comparison of BarA*_Ac_* in *Acidovorax citrulli* AAC00-1 and GacS in *Pseudomonas syringae* pv. *tomato* DC3000. **(B)** The tree was constructed based on amino acid sequences of BarA proteins using the neighbor-joining method in MEGA 7. *Streptomyces glaucescens* was used as an outgroup. The bootstrap values (1,000 replicates) were shown at the nodes. The bar indicates sequence divergence.

The gene *barA_Ac_* is located at the genomic nucleotides 2259155 to 2264023 of the *A. citrulli* group II strain AAC00-1 (GenBank accession number CP000512.1), and encodes 1622 amino acids. The protein it encodes is annotated as a multi-sensor hybrid histidine kinase. It has two transmembrane domains and a signal transduction histidine kinase for regulating the C4-dicarboxylate transport system (COG4191 super family) at its N-terminus, including nifL_nitrog (nifL_nitrog super family) and a PAS domain (PAS super family). In addition, there is a hybrid sensory histidine kinase BarA domain (PRK11107 super family) at the C-terminus (Figure 1A). GacS in *P*. *syringae* pv. *tomato* DC3000 only contains a hybrid sensory histidine kinase BarA domain (PRK11107 super family; Figure 1A). The amino acid sequence alignment showed that the C-terminus of BarA*_Ac_* of *A. citrulli* AAC00-1 and GacS of *P. syringae* pv. *tomato* DC3000 were relatively conserved (Supplementary Figure 1). The amino acid sequence identity of BarA*_Ac_* in *A. citrulli* AAC00-1 and GacS in *P. syringae* pv. *tomato* DC3000 is 36.85%. The STRING database,^6^ a website of an online functional protein association network, shows that T3SS regulators HrpG and HrpX may interact with BarA*_Ac_*. The BarA phylogenetic tree showed that *A. citrulli* AAC00-1 strain (group II) BarA*_Ac_*, *A. citrulli* M6 strain (group I) BarA_1, *A. avenae* ATCC 19860 Acav_3096 belong to the same clade, indicating that they are closely related, while *Pseudomonas* spp. GacS belong to another clade (Figure 1B).

The successful construction of the *barA_Ac_* mutant strain, Δ*barA_Ac_*, was confirmed by PCR amplification of the DNA from strain Δ*barA_Ac_* using 2063-S and 2063-A primers (Table 2) followed by sequencing of the PCR amplicons (data not shown). The complementary strain, ∆*barA_Ac_*comp, showed resistance to kanamycin, indicating that the plasmid, pBBR-*Aave_2063*, was successfully transferred to ∆*barA_Ac_* (Table 1). The presence of pBBR-*Aave_2063* in Δ*barA_Ac_*comp was further confirmed by PCR amplification of template DNA from strain Δ*barA_Ac_*comp with primers 2063-S and 2063-A (Table 2) followed by sequencing of PCR products (data not shown).

### *barA_Ac_* plays an important role in *Acidovorax citrulli* virulence and ability to induce a hypersensitive response in tobacco

**Figure 2:**
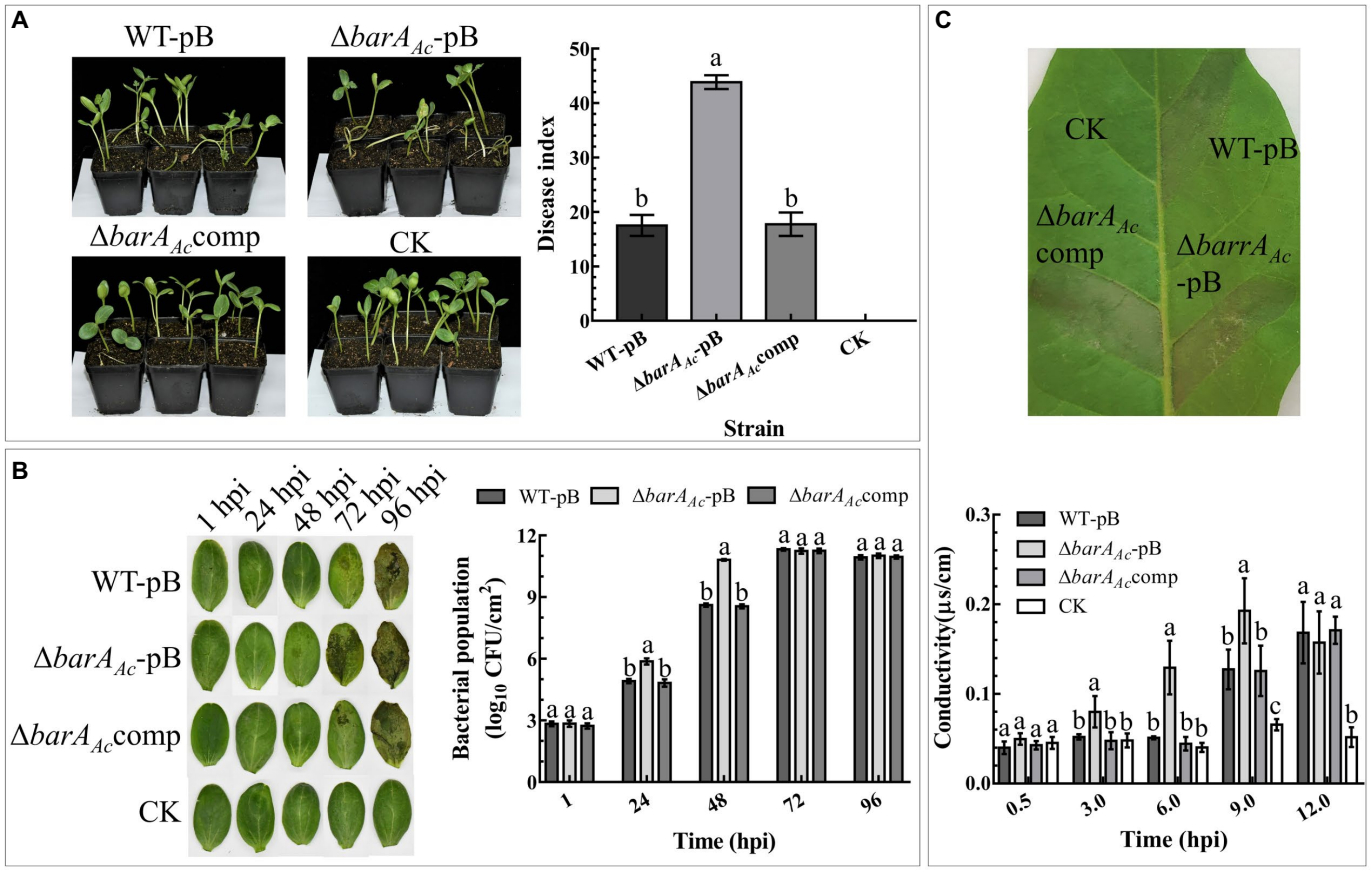
The deletion of *barA_Ac_* affected the virulence, host proliferation, and ability to induce a hypersensitive response (HR) on tobacco (*Nicotiana tabacum*). **(A)** Watermelon seeds were inoculated with *Acidovorax citrulli* wild-type strain WT-pB, Δ*barA_Ac_*-pB, and Δ*barA_Ac_*comp by soaking. After 18 days, the disease indices of the germinated seedlings were determined. Sterilized water was used as a negative control (CK). The bar chart shows the average disease index for 3 replicates, with 14~18 seedlings in each replicate. The error bar represents the standard deviation, and different letters on each treatment indicate significant differences (ANOVA, *p* < 0.05). **(B)** Determination of the bacterial population levels of WT-pB, Δ*barA_Ac_*-pB, and Δ*barA_Ac_*comp in watermelon cotyledons. The data represent the mean ± standard deviation of the bacterial population levels. Different letters on each treatment at each time point indicate significant differences (ANOVA, *p* < 0.05). **(C)** The leaf photo shows the HR induced by test strains on *N. tabacum* leaves. The photo was taken at 24 hpi. The bar chart represents the electrolyte leakage caused by the test strains in *N. tabacum* leaf tissue, and sterile water treatment was used as a negative control (CK). The error bar represents the standard deviation. Different letters on each treatment at each time point indicate significant differences (ANOVA, *p* < 0.05).

To determine the role of *barA_Ac_* in *A. citrulli* virulence, watermelon seeds were inoculated with wild-type strain carrying pBBR1MCS-2 (WT-pB), the mutant strain Δ*barA_Ac_*-pB, and the complementary strain Δ*barA_Ac_*comp. The results showed that the disease index of the mutant strain Δ*barA_Ac_*-pB (mean = 43.83) was significantly higher than WT-pB (mean = 17.51). The average disease index caused by the complementary strain Δ*barA_Ac_*comp was 17.74, similar to WT-pB (Figure 2A).

In order to test whether the enhanced virulence of Δ*barA_Ac_*-pB was related to bacterial proliferation, we compared the population levels of the tested strains in watermelon cotyledons. The results showed that at 1 hpi, there was no significant difference in the bacterial population levels among treatments, indicating that their initial inoculum was consistent. Notably, at 24 hpi and 48 hpi, the population of Δ*barA_Ac_*-pB in the infected leaves was significantly higher than that of WT-pB and Δ*barA_Ac_*comp. However, population levels among tested strains were not significantly different after 72 hpi, indicating that *barA_Ac_* mainly affects the early stages of *A. citrulli* proliferation of watermelon cotyledons (Figure 2B).

To determine whether *barA_Ac_* affects the ability of *A. citrulli* to cause HR on tobacco, strains were infiltrated into tobacco leaves. The results showed that all tested strains caused HR on tobacco (Figure 2C). However, as a measure of the loss of cell integrity, electrolyte leakage in leaves inoculated with Δ*barA_Ac_*-pB was significantly higher than WT-pB and Δ*barA_Ac_*comp (Figure 2C) at 3, 6, and 9 hpi. They were not significantly different at 12 hpi. This indicates that the deletion of *barA_Ac_* accelerated the rate of HR induction.

### *barA_Ac_* affected biofilm formation and swimming motility

**Figure 3:**
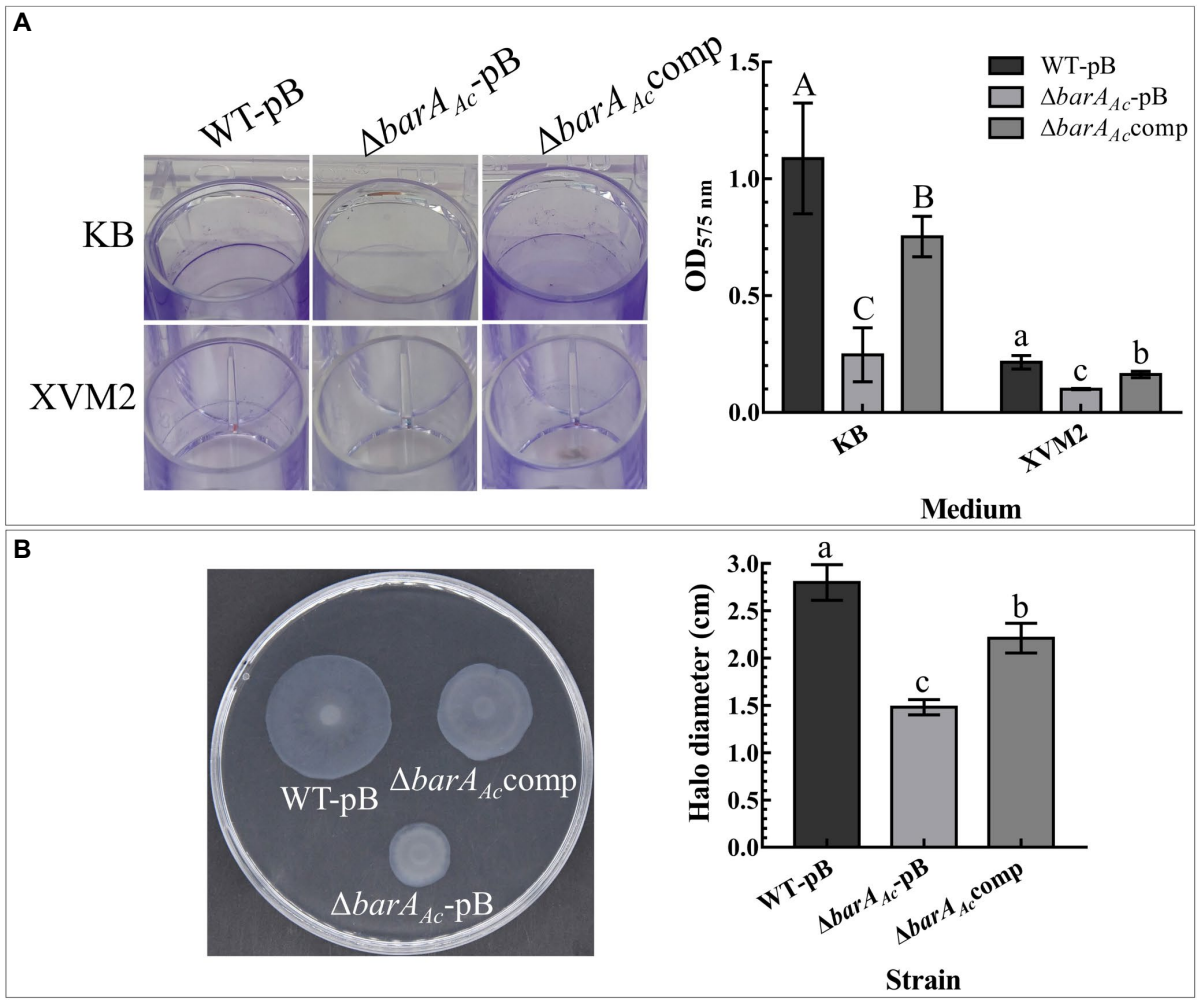
Effects of *barA_Ac_* on biofilm formation, and swimming motility in *Acidovorax citrulli*. **(A)** Biofilms formed by WT-pB, Δ*barA_Ac_*-pB, and Δ*barA_Ac_*comp cultured on polystyrene plates in KB and XVM2 media for 72 h. XVM2 medium served as a medium to simulate the plant environment. The bar chart shows the average OD_575_ absorbance measured after the biofilm was dissolved in 95% ethanol. Each treatment was replicated 6 times. The error bar represents the standard deviation. Different uppercase letters on top of the bar indicate strains with significant differences in biofilm formation ability in KB medium, and different lowercase letters on top of the bar indicate strains with significant differences in biofilmformation ability in XVM2 medium, respectively (ANOVA, *p* < 0.05). **(B)** The swimming motility of WT-pB, Δ*barA_Ac_*-pB, and Δ*barA_Ac_*comp incubated in 0.3% water agar medium for 48 h. The bar chart shows the average halo diameter of the tested strains. Each treatment was replicated 5 times. The error bar represents the standard deviation, and different lowercase letters on top of the bar indicate significant differences (ANOVA, *p* < 0.05).

WT-pB formed a visible biofilm ring on the inner wall of the polystyrene plate when cultured in KB medium. Δ*barA_Ac_*-pB formed very little biofilm, while the complementary strain, Δ*barA_Ac_*comp, formed a visible biofilm ring (Figure 3A). Our quantitative biofilm assay confirmed this observation. The average absorption value of Δ*barA_Ac_-*pB biofilm was significantly lower than WT-pB and Δ*barA_Ac_*comp (Figure 3A). Although the biofilm-forming ability of all the tested strains was reduced when cultured in XVM2 medium relative to KB, the biofilm-forming ability of Δ*barA_Ac_*-pB in XVM2 medium was also significantly lower than WT-pB and Δ*barA_Ac_*comp (Figure 3A).

In the swimming motility assay, the average halo diameter of the mutant strain Δ*barA_Ac_* was significantly lower than WT-pB and the complementary strain Δ*barA_Ac_*comp (*p* < 0.05; Figure 3B). The fact that the halo size for the mutant strain Δ*barA_Ac_* was reduced after 48 hpi indicates impaired swimming motility.

### RNA-seq reveals that the *barA_Ac_* gene plays an important role in the deployment of T3SS in *Acidovorax citrulli*

**Figure 4:**
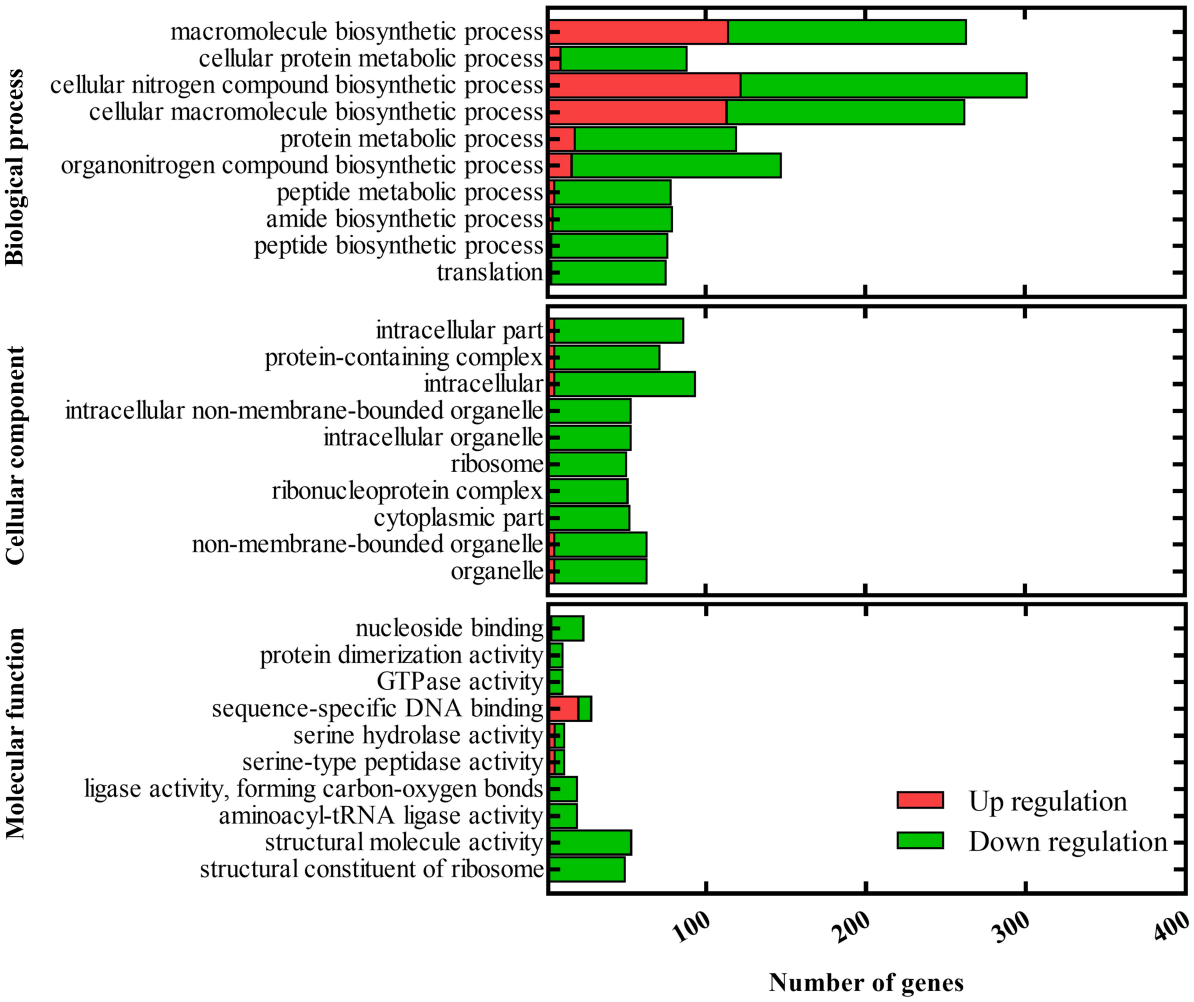
Gene ontology (GO) of differentially expressed genes between the *barA_Ac_* deletion mutant strain Δ*barA_Ac_* and the wild-type strain Aac5. GO analysis takes *p* < 0.05 as the threshold for significant enrichment. The 30 most significant terms are shown. The horizontal axis is the number of differentially expressed genes, and the vertical axis is the GO term.

Since the deletion of *barA_Ac_* affects numerous virulence phenotypes of *A. citrulli* Aac5, we further explored the role of *barA_Ac_* in transcriptional regulation by analyzing RNA-seq data of Δ*barA_Ac_* and wild-type strain Aac5. Compared with the wild-type strain Aac5, a total of 3,332 genes were differentially expressed in Δ*barA_Ac_*, of which 1,641 genes were upregulated and 1,691 genes were downregulated. The RNA-seq results were validated in RT-qPCR experiments with a set of 10 selected genes (Supplementary Figure 2). GO analysis divided the differentially expressed genes (DEGs) into biological process, cellular component, and molecular function (Figure 4). Under the biological process, many genes were downregulated, including translation (72 genes were downregulated while only 3 genes were upregulated), peptide biosynthetic process (73 genes were downregulated while only 3 genes were upregulated), amide biosynthetic process (75 genes were downregulated while only 4 genes were upregulated), peptide metabolic process (73 genes were downregulated while only 5 genes were upregulated), and protein metabolic process (101 genes were downregulated while only 18 genes were upregulated). Under the cellular component, DEGs of cytoplasmic part (*n* = 52), ribonucleoprotein complex (*n* = 51), and ribosome (*n* = 50) were all downregulated. Correspondingly, DEGs involved in structural constituent of ribosome (*n* = 49) in molecular function were all downregulated. Our KEGG pathway analysis showed that pathways of ribosome and oxidative phosphorylation were significantly enriched. In addition, some pathways were also enriched, but not significantly, such as carbon metabolism, aminoacyl-tRNA biosynthesis, microbial metabolism in diverse environments, nicotinate and nicotinamide metabolism, pentose phosphate pathway, biotin metabolism, propanoate metabolism, and bacterial secretion system, etc.

Notably, under the *in vitro* condition, the results of RNA-seq showed that many T3SS-related genes including *hrpX* were upregulated in mutant Δ*barA_Ac_*, while the expression level of *hrpG* was not (Table 3). Type III effectors (T3Es) of phytopathogens play an important role in combating plant defense responses (Block et al., 2008). RNA-seq revealed that the expression of many of the previously predicted T3Es was upregulated in the mutant Δ*barA_Ac_* compared with the wild-type strain Aac5 (Table 4), which further confirmed that *A. citrulli* Aac5 strain *barA_Ac_* plays an important role in the regulation of T3SS.

**Table 3:** Differentially expressed type III secretion system genes in *Acidovorax citrulli* Δ*barA_Ac_* compared to wild-type strain Aac5.

Gene ID	Gene product description	logFC	Adjusted *p-*values
*Aave_0445*	Response regulator receiver protein, HrpG	−0.24	8.98e-3
*Aave_0444*	Transcriptional regulator, AraC family, HrpX	0.88	2.27e-11
*Aave_0447*	Type III secretion regulatory protein HpaA	0.92	7.05e-8
*Aave_0449*	Type III secretion protein R	0.81	1.15e-3
*Aave_0450*	Type III secretion protein Q	1.49	1.81e-18
*Aave_0451*	Type III secretion regulatory protein HpaP	1.92	2.42e-7
*Aave_0452*	Type III secretion protein V	1.18	7.33e-18
*Aave_0453*	Type III secretion protein U	1.16	6.39e-11
*Aave_0454*	Type III secretion protein HrpB7	1.53	7.27e-11
*Aave_0455*	Type III secretion protein T	0.83	3.85e-6
*Aave_0456*	Type III secretion protein HrpY	1.15	4.23e-17
*Aave_0457*	Type III secretion protein HrpW	1.20	5.57e-12
*Aave_0463*	ATP synthase in type III secretion protein N	0.99	1.27e-11
*Aave_0464*	Type III secretion protein E	1.10	2.17e-10
*Aave_0465*	Type III secretion protein B4	1.79	1.43e-16
*Aave_0466*	Type III secretion protein J	0.91	5.44e-7
*Aave_0473*	Type III secretion protein D	1.14	3.20e-12
*Aave_0474*	Type III secretion protein C	1.24	7.08e-20
*Aave_0479*	Type III secretion protein HrpB1	1.01	7.34e-4

Gene ID, the locus tags of differentially expressed genes identified by hits in a Blastn search against the A. citrulli AAC00-1 genome (NC_008752); FC, fold change.

**Table 4:** Differentially expressed type III effector genes in *Acidovorax citrulli* Δ*barA_Ac_* compared to wild-type strain Aac5.

Gene ID	Gene product description	logFC	Adjusted *p*-values
*Aave_3452* ^a^	AvrPphE homolog	0.80	1.27e-7
*Aave_3462* ^a^	YopP/AvrRxv homolog	1.19	9.98e-16
*Aave_4728* ^a^	Hypothetical protein	1.10	1.78e-13
*Aave_2177* ^b^	Hypothetical protein	0.80	4.76e-6
*Aave_1555* ^b^	Hypothetical protein	0.85	5.47e-6
*Aave_3960* ^b^	Hypothetical protein	0.87	5.01e-4
*Aave_3626* ^c^	XopQ homolog	0.70	1.33e-5
*Aave_3847* ^c^	Hypothetical protein	0.98	2.81e-9
*Aave_4254* ^c^	XopAE homolog	1.10	2.58e-11
*Aave_4472* ^c^	XopF1 homolog	1.23	7.16e-21
*Aave_4606* ^c^	RipAY homolog	1.37	1.82e-24
*Aave_4612* ^c^	XopR homolog	1.26	2.49e-14
*Aave_4631* ^c^	Leucine-rich repeat protein	1.19	2.60e-14
*Aave_4632* ^c^	Hypothetical protein	0.92	3.67e-13
*Aave_0433* ^c^	XopK homolog	0.99	9.46e-15
*Aave_0458* ^c^	Effector GALA homolog	1.47	4.81e-20
*Aave_1090* ^c^	Effector AWR homolog	1.44	4.52e-23
*Aave_1520* ^c^	HopBF1 homolog	0.72	6.12e-4
*Aave_3085* ^c^	XopV homolog	0.91	2.84e-6
*Aave_2844* ^c^	Type III effector, lipase domain	0.56	1.18e-3

Gene ID, the locus tags of differentially expressed genes (DEGs) identified by hits in a Blastn search against the *A. citrulli* AAC00-1 genome (NC_008752); FC, fold change.

a The DEGs whose products are annotated as T3Es in *A. citrulli* strain AAC00-1 (Eckshtain-Levi et al., 2014).

b The homologs of these hypothetical proteins have been validated as T3Es in *A. citrulli* strain M6 by experimental assays (Jiménez-Guerrero et al., 2020).

c The gene product is homologous to known T3Es by BlastP analysis (Jiménez-Guerrero et al., 2020).

### *barA_Ac_* inhibited the protein expression of the T3SS regulator HrpG

**Figure 5:**
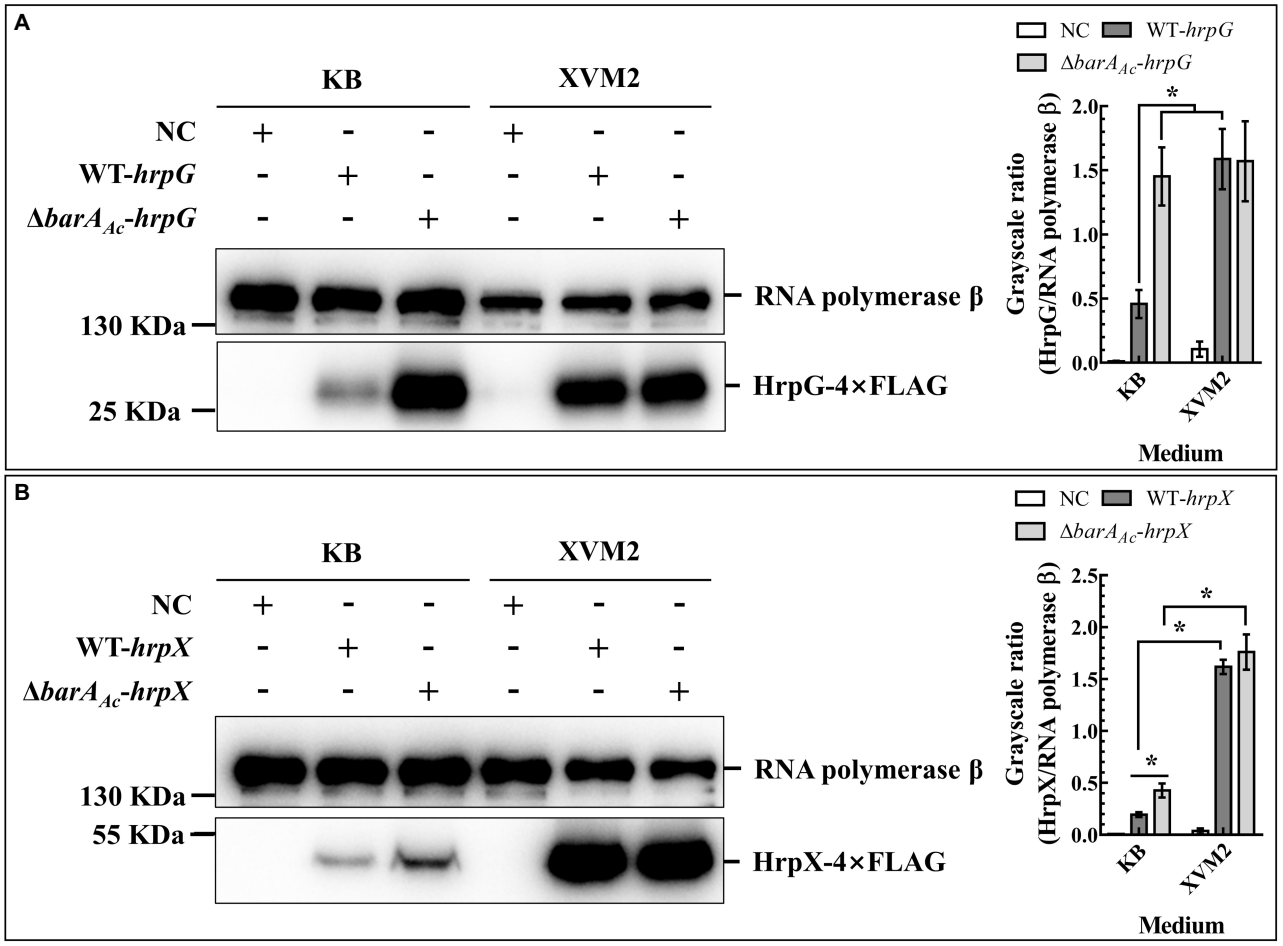
The deletion of *barA_Ac_* enhanced the expression of HrpG and HrpX in *Acidovorax citrulli*. The expression of HrpG **(A)** and HrpX **(B)** detected by Western blot in the wild-type strain WT-*hrpG* and the *barA_Ac_* deletion mutant strain Δ*barA_Ac_*-*hrpG* carrying pBBR-*hrpG*-4Flag, and the wild-type strain WT-*hrpX* and the *barA_Ac_* deletion mutant strain Δ*barA_Ac_*-*hrpX* carrying pBBR-*hrpX*-4Flag in King’s B medium and XVM2 medium. The negative control (NC) was the wild-type strain Aac5 carrying pBBRNolac-4Flag. RNA polymerase β was used as a control for protein loading. The bar chart shows the quantitative protein expression levels (the mean value of the grayscale ratios) calculated using Image J (National Institutes of Health, United States). The error bar represents the standard deviation, and ^*^indicates significant difference between treatments (*t*-test, *p* < 0.05).

HrpG and HrpX are the key regulators of T3SS in *A. citrulli* (Zhang et al., 2018). To determine whether *barA_Ac_* affects T3SS deployment by regulating the expression of HrpG or HrpX, total proteins of Δ*barA_Ac_*-*hrpG* and WT-*hrpG* were extracted under two culture conditions (KB and XVM2). HrpG expression of Δ*barA_Ac_*-*hrpG* was significantly higher than that of WT-*hrpG* when cultured in KB medium, while the gap between them was narrowed when cultured in XVM2 medium (Figure 5A). HrpG can act on the *hrpX* promoter and regulate *hrpX* expression (Zhang et al., 2018). When cultured in KB medium, the HrpX protein expression of Δ*barA_Ac_*-*hrpX* was significantly higher than that of WT-*hrpX*, while when cultured in XVM2 medium, there was no significant difference between them (Figure 3B). These results indicate that *barA_Ac_* inhibited the expression of T3SS regulators HrpG and HrpX in KB medium.

### *barA_Ac_* regulated *hrpX*, but not *hrpG* at the transcription level

In order to determine if *barA_Ac_* affected the expression of HrpG and HrpX at the transcriptional level, the promoter activity of *hrpG* and *hrpX* of the test strain was determined. *hrpG* promoter activity of Δ*barA_Ac_*-*hrpG*p-GUS did not change significantly compared with WT-*hrpG*p-GUS, regardless of whether they were cultured in KB or XVM2 medium (Figure 5A). However, regardless of the media, the *hrpX* promoter activity of Δ*barA_Ac_*-*hrpX*p-GUS was significantly higher than that of WT-*hrpX*p-GUS (Figure 5B), which is consistent with the RNA-seq result.

## Discussion

The disruption of T3SS in *A*. *citrulli* resulted in a complete loss of virulence on host plants and the ability to induce an HR on tobacco (Zhang et al., 2018), suggesting that T3SS plays a pivotal role in *A. citrulli* pathogenicity. In our study, the deletion of *barA_Ac_* led to enhanced virulence in host tissues and accelerated HR in tobacco relative to the wild-type strain (Figure 1). Based on RNA-seq data (Table 3) and the important contribution of T3SS in pathogenic bacteria, we speculate that this may be due to the association between BarA*_Ac_* and the T3SS.

**Figure 6:**
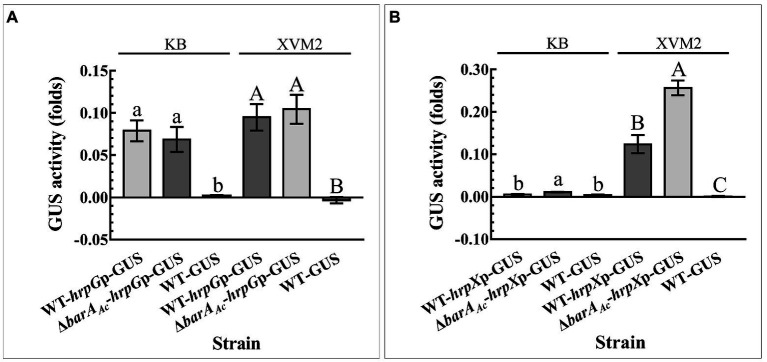
The deletion of *barA_Ac_* enhanced *hrpX* promoter activity. The activity of β-Glucuronidase (GUS) was determined by **(A)** the wild-type strain WT-*hrpG*p-GUS and the *barA_Ac_* deletion mutant strain Δ*barA_Ac_*-*hrpG*p-GUS carrying pBBR-GUS-*hrpG*p, and **(B)** WT-*hrpX*p-GUS and Δ*barA_Ac_*-*hrpX*p-GUS carrying pBBR-GUS-*hrpX*p in King’s B (KB) medium and XVM2 medium that simulates plant environment. The wild-type strain WT-GUS carrying pBBRNolacGUS was used as a negative control. The error bar represents the standard deviation. Different lowercase and uppercase letters on top of the bar indicate strains with significant differences in the GUS activity in KB and XVM2 medium, respectively (ANOVA, *p* < 0.05).

Consistent with the hypothesized association, we showed that the deletion of *barA_Ac_* enhanced the expression of the HrpG protein (Figure 6), but not *hrpG* promoter activity and transcriptional levels of *hrpG* (Table 3; Figure 6). Notably, the enhancement of HrpG expression in *barA_Ac_* deletion mutant became less obvious in the XVM2 induction medium. We speculate that this was because the inhibitory effect of *barA_Ac_* on HrpG is weakened during *in vivo* plant infection. It is possible that BarA*_Ac_* has a role in the conversion between saprophytic and pathogenic states. For example, when *P. syringae* enters the leaf tissue, it changes from motile to sessile, and begins to secrete effectors into the host cell through the T3SS (Abramovitch et al., 2006; Schreiber and Desveaux, 2011; Xin et al., 2018). In *A. citrulli*, the *barA_Ac_* mutant strain enhanced the expression of HrpG and reduced swimming motility when cultured *in vitro*, indicating that BarA*_Ac_* may inhibit T3SS and promote swimming motility during the saprophytic phase of *A. citrulli*. This is different from GacS in *P. aeruginosa*, which negatively regulated swimming motility (Valentini et al., 2017). This indicates BarA*_Ac_* from *A. citrulli* and GacS from *P. aeruginosa* may have different regulons. In addition, RNA-seq showed that for genes involved in translation in biological processes, 73 and 2 genes were downregulated and upregulated, respectively. For genes involved in the peptide biosynthetic process, only 3 genes were upregulated, and 73 genes were downregulated in mutant strain compared with wild-type Aac5, suggesting that synthesis of other proteins was decreased while T3SS deployment was increased in Δ*barA_Ac_*. This implies that BarA*_Ac_* may play a tradeoff role between virulence and metabolism in *A. citrulli* Aac5.

Notably, when cultured in KB medium, the protein expression of HrpX was not upregulated as much as that of HrpG in the *barA_Ac_* deletion mutant Δ*barA_Ac_* compared with the wild-type strain (Figure 5). In other words, HrpX is not abundantly expressed under nutrient-rich conditions in response to the massive accumulation of HrpG in *A. citrulli* Aac5, although it has been previously proved that HrpG can activate the expression of *hrpX* in *A. citrulli* Aac5 (Zhang et al., 2018). In *Xanthomonas citri* subsp. *citri* (*Xcc*), phosphorylation of HrpG is required for its full transcriptional activation activity (Andrade et al., 2014). HrpG retains its DNA binding activity but loses its ability to activate transcription after aspartic acid of the 61th position is replaced in *Xcc* (Andrade et al., 2014). In fact, when we replaced the 52nd or 60th aspartic acid of HrpG in *A*. *citrulli* Aac5, Aac5 lost its ability to induce HR on tobacco, while the replacement of the 46^th^ aspartic acid of HrpG in Aac5 did not affect its ability to induce HR on tobacco (data not shown). This suggests that aspartic acid at the 52nd or 60th position of HrpG in *A*. *citrulli* Aac5 may be its potential phosphorylation site. We speculate that *A. citrulli* Aac5 may have a mechanism similar to *Xcc*, that is, in a nutrient-rich environment, the HrpG protein would not be phosphorylated or the proportion of phosphorylated HrpG would be low, so as to achieve the purpose of shutting down the expression of T3SS. This inhibition seems to be necessary based on the fact that the deployment of T3SS consumes a large amount of energy (Teper et al., 2021). We speculate that in a nutrient-poor environment similar to the host plant apoplast, HrpG is heavily phosphorylated, which turns on its transcriptional activation activity and T3SS-related genes will be expressed in large quantities. However, histidine kinases that may be involved in HrpG phosphorylation have been reported only in *Xanthomonas axonopodis* pv. *citri* and *Xanthomonas campestris* pv. *campestris* (Alegria et al., 2004; Li et al., 2014).

T3SS plays an important role in the early proliferation of host plants by phytopathogens (de Bernonville et al., 2014; Hu et al., 2014; Zhou et al., 2015). The population of the Δ*barA_Ac_* mutant strain was significantly higher than that of the wild-type strain at 24 hpi, which may be due to the rapid deployment of T3SS in the Δ*barA_Ac_* strain. At the later stage of proliferation (72 hpi), there was no significant difference in the population levels between the seedlings inoculated with Δ*barA_Ac_* and the wild-type strain. We speculate that although the deployment of T3SS in the wild-type strain was slower than that of Δ*barA_Ac_*, the deployment of T3SS in the wild-type strain gradually increased over time. A similar phenomenon occurred for the tobacco HR assay.

Swimming motility of bacteria is driven by flagella (Macnab, 2004). In this study, RNA-seq data showed that 13 flagella-related genes were upregulated and 13 were downregulated in the *barA_Ac_* mutant strain compared with the wild-type strain Aac5 (Supplementary Table 3). Among them, the downregulated genes include flagellar structure genes, such as FliQ, a flagellar biosynthetic protein. Therefore, we speculated that the reduced swimming motility of the *barA_Ac_* mutant strain may be caused by the incomplete expression of flagellar proteins. Furthermore, in order to characterize the effect of BarA*_Ac_* on other virulence associated phenotypes, we measured biofilm formation of the mutant strain. The formation of biofilm is critical for the virulence of certain plant pathogens (Dow et al., 2003; Fujishige et al., 2006). The phytopathogenic bacterium *X. axonopodis* pv*. citri* showed reduced virulence when it was unable to produce biofilm (Rigano et al., 2007). However, we observed that biofilm production of the *barA_Ac_* mutant was significantly reduced while virulence was enhanced. Similarly, the *acrR* mutant (a global regulatory gene) in *A. citrulli* strain Aac5, displayed enhanced biofilm formation but reduced virulence (Guan et al., 2020).

**Figure 7:**
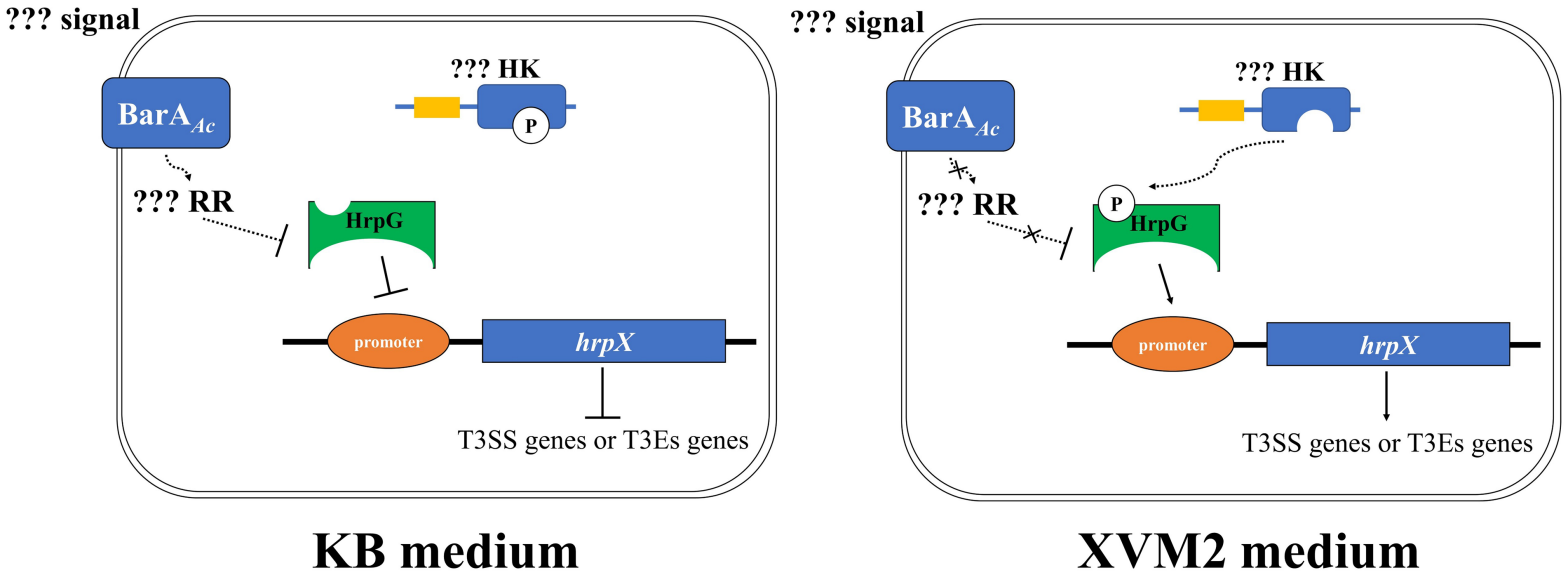
The regulation model of BarA*_Ac_* in *Acidovorax citrulli* Aac5 strain. Signal refers to extracellular signal that BarA*_Ac_* can perceive and RR refers to response regulator regulated by BarA*_Ac_*, respectively. HK refers to the histidine kinase possibly involved in HrpG phosphorylation, and promoter refers to the promoter region of *hrpX* gene. The solid line indicates direct regulation, the dashed line indicates unknown pathway or indirect regulation, the blunt arrow indicates inhibition, and the pointed arrow indicates activation. In addition, the curve indicates the transfer of the phosphate group.

In conclusion, our results indicate that BarA*_Ac_* acts as a negative regulator of virulence in *A. citrulli* group II strain Aac5. We propose a model (Figure 7) in which BarA*_Ac_* would negatively regulate HrpG at the protein level by regulating downstream unknown response regulator when *A*. *citrulli* Aac5 is cultured in KB medium. At the same time, HrpG could not activate *hrpX* completely at the transcriptional level due to the lack of phosphorylation or low phosphorylation rate, so as to limit the expression of *hrpX* and downstream T3SS genes of Aac5 in KB medium to the maximum extent. When Aac5 is cultured in XVM2 medium, HrpG protein can be expressed in large quantities. At the same time, the upstream unknown HK can transfer phosphate groups to HrpG, which can phosphorylate HrpG in large quantities and exert transcriptional activation activity, and activate the downstream *hrpX* and T3SS genes (Figure 7). However, many aspects of this model are unknown, and future studies on these aspects will be of great significance to supplement the regulatory network of the pathogenicity mechanisms in *A*. *citrulli*.

## Data availability statement

The raw data supporting the conclusions of this article will be made available by the authors, without undue reservation.

## Author contributions

PQ, MZ, and TZ designed the research and wrote the paper. PQ executed the experiments. MZ, YYe, YYa, and WG performed the data analyses. PQ, RW, TZ, and MZ critically reviewed the manuscript. All authors contributed to the article and approved the submitted version.

## Funding

This study was supported by the China Earmarked Fund for Modern Agro-industry Technology Research System (CARS-25), the National Natural Science Foundation of China (31701754), the National Key Research and Development Program of China (2018YFD0201300), Central Public-Interest Scientific Institution Basal Research Fund (S2022XM25), the Agricultural Science and Technology Innovation Program of the Chinese Academy of Agricultural Sciences (CAAS-ASTIP), the Xinjiang Production and Construction Corps’ Scientific and Technological Research Plan Project in Agri-cultural (2022AB015), and the Key Special Project of Revitalize Inner Mongolia with Science and Technology (NMKJXM202107-03).

## Conflict of interest

The authors declare that the research was conducted in the absence of any commercial or financial relationships that could be construed as a potential conflict of interest.

## Publisher’s note

All claims expressed in this article are solely those of the authors and do not necessarily represent those of their affiliated organizations, or those of the publisher, the editors and the reviewers. Any product that may be evaluated in this article, or claim that may be made by its manufacturer, is not guaranteed or endorsed by the publisher.

## Supplementary material

The Supplementary material for this article can be found online at: https://www.frontiersin.org/articles/10.3389/fmicb.2022.1064577/full#supplementary-material

## Footnotes

^1^ https://www.ncbi.nlm.nih.gov/Structure/cdd/wrpsb.cgi

^2^ https://string-db.org/

^3^ https://www.genome.jp/tools-bin/clustalw

^4^ https://www.genome.jp/kegg/

^5^ http://www.simgene.com/Primer3

^6^ https://string-db.org/
