# Supplementary material for: A putative multi-sensor hybrid histidine kinase, BarAAc, inhibits the expression of the type III secretion system regulator HrpG in Acidovorax citrulli
Source: Front Microbiol. 2022 Nov 30;13:1064577. doi: 10.3389/fmicb.2022.1064577 (PMC9748350; doi:10.3389/fmicb.2022.1064577)
Supplement: Supplementary file 1 [file Table_1.DOCX]

**Supplementary Table 1 Protein information for constructing the phylogenetic tree**

| **Gene ID** | **Protein description** | **Organism** |
| --- | --- | --- |
| *Aave_2063* | Multi-sensor hybrid histidine kinase | *Acidovorax citrulli* AAC00-1 |
| *APS58_0264* | Multi-sensor hybrid histidine kinase, BarA_1 | *Acidovorax citrulli* M6 |
| *Acav_3096* | Multi-sensor hybrid histidine kinase | *Acidovorax avenae* subsp. *avenae* ATCC 19860 |
| *PA0928* | Sensor histidine kinase BarA, GacS | *Pseudomonas aeruginosa* PAO1 |
| *PSF113_4428* | Sensor histidine kinase, BarA / GacS | *Pseudomonas ogarae* |
| *PSPPH_3719* | Sensor histidine kinase BarA, GacS | *Pseudomonas savastanoi* pv. *phaseolicola* 1448A |
| *PSPTO_1691* | Sensor histidine kinase BarA, GacS | *Pseudomonas syringae* pv. *tomato* DC3000 |
| *CSG_14150* | BarA sensory histidine kinase (= VarS = GacS) | *Campylobacter fetus* subsp. *venerealis* 84-112 |
| *SGLAU_01010* | GacS | *Streptomyces glaucescens* |
