# Supplementary material for: A putative multi-sensor hybrid histidine kinase, BarAAc, inhibits the expression of the type III secretion system regulator HrpG in Acidovorax citrulli
Source: Front Microbiol. 2022 Nov 30;13:1064577. doi: 10.3389/fmicb.2022.1064577 (PMC9748350; doi:10.3389/fmicb.2022.1064577)
Supplement: Supplementary file 2 [file Table_2.DOCX]

**Supplementary Table 2 Primers used for RT-qPCR**

| **Primers** | **Sequence (5’-3’)** | **Product of PCR amplicon (bp)** |
| --- | --- | --- |
| 0450-S | CCGAAATCGCCCTCCAC | 158 |
| 0450-A | GATGTCGGTCAGCGGCAG |  |
| 0465-S | CCGACCATGTCGATCCCA | 123 |
| 0465-A | AGCGGCGAACGAACCAG |  |
| 0474-S | CCGCATCAAGGGATTCAGC | 121 |
| 0474-A | CGGGCCGGACACATAGAT |  |
| 0475-S | GGCAACGACTTCAATCCC | 130 |
| 0475-A | AATCCATCATCGGCAGGT |  |
| 1090-S | CTGGAGGGCAGCGAAGAT | 84 |
| 1090-A | CAGCGGGGTGGAGAGGT |  |
| 1619-S | CACCATTCGCTGGCTCG | 112 |
| 1619-A | CGGAATCTGCGTGGTCAA |  |
| 2552-S | TCGAAGCGTCGGTGGGA | 121 |
| 2552-A | GCGAGCAGCAGTTTGAAGG |  |
| 3011-S | CTTCGCCCAGCACAGCC | 111 |
| 3011-A | TCCACGTTCATGAGCCGC |  |
| 3744-S | CATGGCCGAACTGGTCTT | 110 |
| 3744-A | GCGAAGGCTGGTATGGG |  |
| 4760-S | GGCGGAACCGACACCAT | 155 |
| 4760-A | TAGTAGCCCCGCTTGTGC |  |
