# Supplementary material for: A putative multi-sensor hybrid histidine kinase, BarAAc, inhibits the expression of the type III secretion system regulator HrpG in Acidovorax citrulli
Source: Front Microbiol. 2022 Nov 30;13:1064577. doi: 10.3389/fmicb.2022.1064577 (PMC9748350; doi:10.3389/fmicb.2022.1064577)
Supplement: Supplementary file 3 [file Table_3.DOCX]

**Supplementary Table 3 Differentially expressed genes related to the flagellar assembly in *A.citrulli*.**

| **Gene ID** | **Gene product description** | **logFC** | **Adjusted *p* value** |
| --- | --- | --- | --- |
| *Aave_4396* | Flagellar hook-basal body complex subunit FliE | 0.48 | 6.79e-3 |
| *Aave_4394* | Flagellar motor switch protein FliG | -0.36 | 8.22e-3 |
| *Aave_4392* | Flagellar protein export ATPase FliI | 0.53 | 1.89e-5 |
| *Aave_4391* | Flagellar export protein FliJ | 0.63 | 2.29e-3 |
| *Aave_4390* | Flagellar hook-length control protein | 0.57 | 8.69e-6 |
| *Aave_4389* | Flagellar basal body-associated protein FliL | -0.28 | 1.51e-2 |
| *Aave_4388* | Flagellar motor switch protein FliM | -0.43 | 1.25e-4 |
| *Aave_4387* | Flagellar motor switch protein FliN | -0.48 | 3.26e-4 |
| *Aave_4384* | Flagellar biosynthetic protein FliQ | -0.78 | 3.13e-3 |
| *Aave_4413* | Flagellar biosynthesis protein FlhA | 0.27 | 1.72e-2 |
| *Aave_4419* | Flagellar basal body P-ring formation protein FlgA | 0.93 | 2.61e-9 |
| *Aave_4420* | Flagellar basal-body rod protein FlgB | 0.53 | 1.18e-3 |
| *Aave_4424* | Flagellar basal-body rod protein FlgF | 0.51 | 1.67e-4 |
| *Aave_4426* | Flagellar L-ring protein | 0.57 | 4.90e-5 |
| *Aave_4428* | Flagellar P-ring protein | 0.42 | 1.40e-3 |
| *Aave_4429* | Flagellar rod assembly protein/muramidase FlgJ | 0.34 | 1.27e-2 |
| *Aave_4401* | Flagellin domain protein | -0.41 | 3.83e-3 |
| *Aave_4400* | Flagellin domain protein | -0.46 | 2.16e-5 |
| *Aave_4399* | Flagellar hook-associated 2 domain protein | -0.25 | 3.90e-3 |
| *Aave_4397* | Flagellar protein FliT | -0.51 | 1.20e-4 |
| *Aave_4408* | MotA/TolQ/ExbB proton channel | -0.52 | 1.06e-4 |
| *Aave_4418* | Anti-sigma-28 factor, FlgM | -0.29 | 1.34e-2 |
| *Aave_1406* | RNA polymerase, sigma 70 subunit, RpoD | -0.99 | 2.93e-24 |
| *Aave_2006* | Flagellar transcriptional activator, FlhD subunit | 1.13 | 9.54e-13 |
| *Aave_2005* | Flagellar transcriptional activator, FlhC subunit | 0.52 | 3.58e-4 |
| *Aave_4416* | RNA polymerase sigma factor FliA | -0.43 | 5.27e-4 |

Differentially expressed genes related to flagellar assembly in Δ*barA_Ac_* compared to wild-type strain Aac5 were listed in the table. Gene ID: the locus tags of differentially expressed genes that identified by hits in a Blastn search against the *A. citrulli* AAC00-1 genome (NC_008752). FC: fold change.
