# Supplementary figures and images for: A putative multi-sensor hybrid histidine kinase, BarAAc, inhibits the expression of the type III secretion system regulator HrpG in Acidovorax citrulli
Source: Front Microbiol. 2022 Nov 30;13:1064577. doi: 10.3389/fmicb.2022.1064577 (PMC9748350; doi:10.3389/fmicb.2022.1064577)

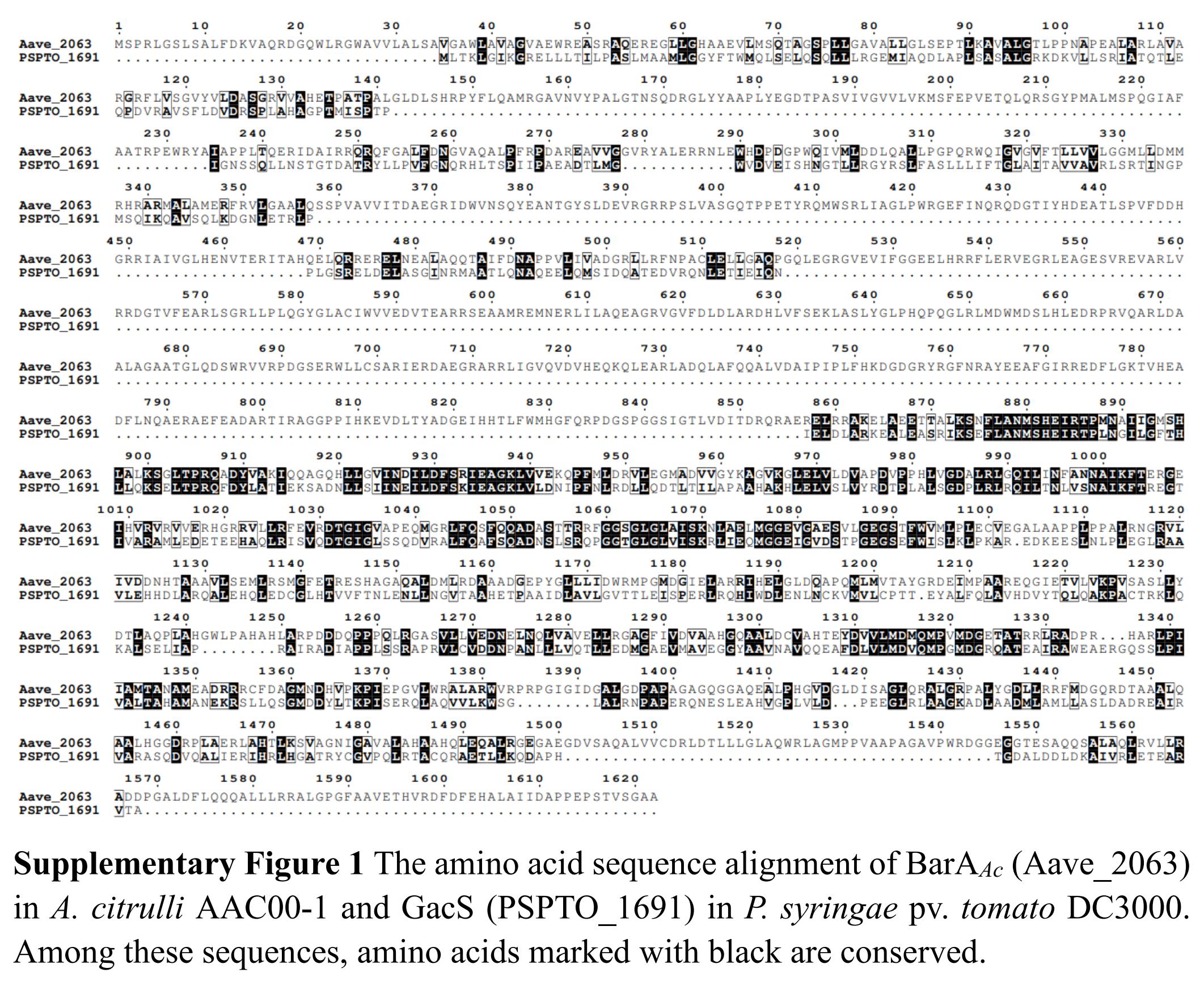

Supplement: Supplementary file 4 [file Image_1.JPEG]

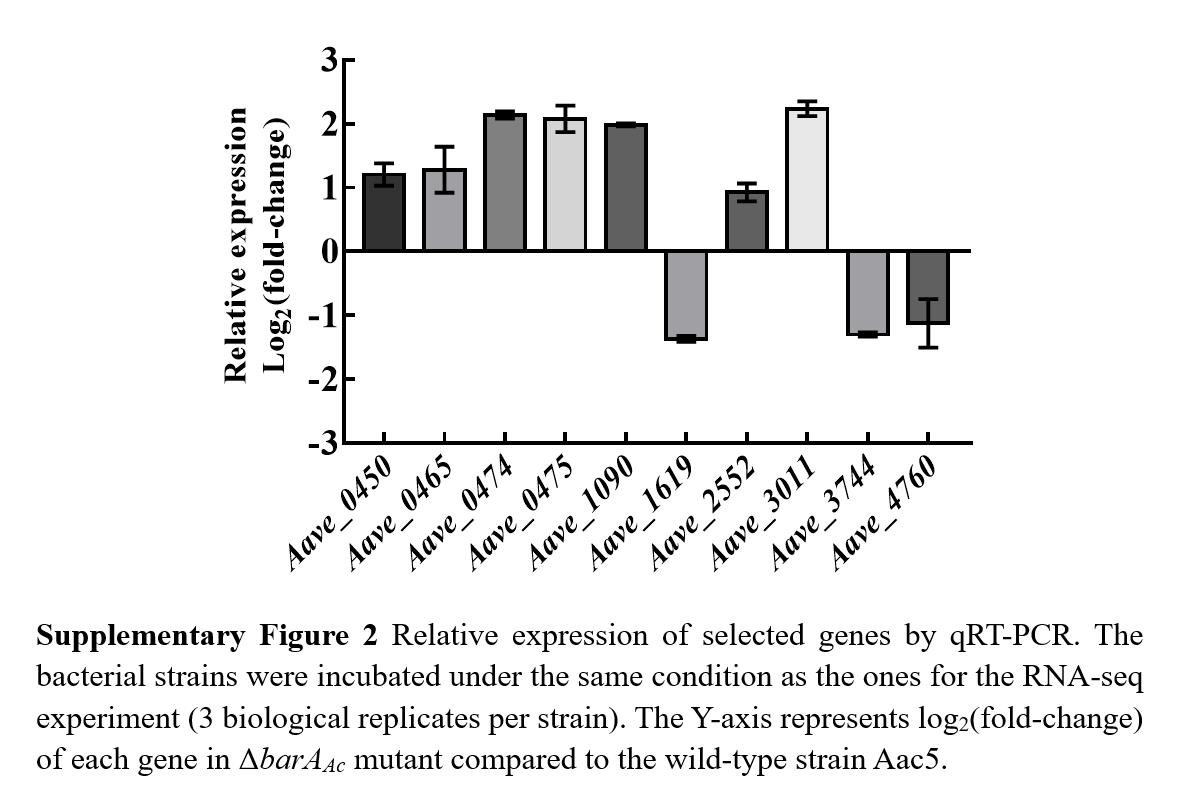

Supplement: Supplementary file 5 [file Image_2.JPEG]
